# Effects of Different Types of Exercise on Bone Mineral Density in Postmenopausal Women: A Systematic Review and Meta-analysis

**DOI:** 10.1007/s00223-020-00744-w

**Published:** 2020-08-12

**Authors:** Wolfgang Kemmler, Mahdieh Shojaa, Matthias Kohl, Simon von Stengel

**Affiliations:** 1 Institute of Medical Physics, Friedrich-Alexander University Erlangen-Nürnberg, Henkestrasse 91, 91052 Erlangen, Germany; 2 Department of Medical and Life Sciences, University of Furtwangen, Schwenningen, Germany

**Keywords:** Bone mineral density, Exercise, Weight bearing exercise, Resistance exercise, Postmenopausal women

## Abstract

In this sub-analysis of a comprehensive meta-analysis, we aimed to determine the effect of different types of exercise on (areal) bone mineral density (BMD) in postmenopausal women. A systematic review of the literature according to the PRISMA statement included (a) controlled trials, (b) with at least one exercise and one control group, (c) intervention ≥ 6 months, (d) BMD assessments at lumbar spine (LS), femoral neck (FN) or total hip (TH), (e) in postmenopausal women. Eight electronic databases were scanned without language restrictions up to March 2019. The present subgroup analysis was conducted as a mixed-effect meta-analysis with “type of exercise” as the moderator. The 84 eligible exercise groups were classified into (a) weight bearing (WB, *n* = 30) exercise, (b) (dynamic) resistance exercise (DRT, *n* = 18), (c) mixed WB&DRT interventions (*n* = 36). Outcome measures were standardized mean differences (SMD) for BMD-changes at LS, FN and TH. All types of exercise significantly affect BMD at LS, FN and TH. SMD for LS average 0.40 (95% CI 0.15–0.65) for DRT, SMD 0.26 (0.03–0.49) for WB and SMD 0.42 (0.23–0.61) for WB&DRT. SMD for FN were 0.27 (0.09–0.45) for DRT, 0.37 (0.12–0.62) for WB and 0.35 (0.19–0.51) for WB&DRT. Lastly, SMD for TH changes were 0.51 (0.28–0.74) for DRT, 0.40 (0.21–0.58) for WB and 0.34 (0.14–0.53) for WB&DRT. In summary, we provided further evidence for the favorable effect of exercise on BMD largely independent of the type of exercise. However, in order to generate dedicated exercise recommendations or exercise guideline, meta-analyses might be a too rough tool.

## Introduction

Exercise is considered a highly relevant component in the prevention and treatment of osteoporosis and fracture reduction [1, 2]. Consequently numerous exercise studies (review in [3]) aim to increase bone strength, predominately assessed by (areal) bone mineral density (BMD) in postmenopausal women, as the most prominent and largest cohort at risk for osteoporosis. However, although there are some evidence-based recommendations for exercise protocols [1, 4, 5], the most promising exercise to address BMD still remains unsettled [2]. Apart from exercise parameters and principles, even basic decisions, for example about the type of exercise that should be applied, is still (or once again) controversial [6, 7]. In a recent meta-analysis, Rahimi et al. [6] reported the absence of effects of resistance exercise and negative effects of weight bearing aerobic exercise on BMD at lumbar spine (LS) and femoral neck (FN) in postmenopausal cohorts 60 years and older (*n* = 16). Provided that these data are reliable and generalizable to the entire cohort of postmenopausal women, all the current exercise recommendations (e.g., [1, 4, 5, 8, 9]. and—even more importantly—the exercise effect on BMD in general are rendered questionable. In order to verify the findings of Rahimi et al. [6], and to estimate the effects of different roughly classified types of exercise on BMD at different regions of interest (ROI), we conducted a sub-analysis based on a recent comprehensive meta-analysis on exercise effects on BMD in postmenopausal women [3]. Similarly to Rahimi et al. [6], we roughly categorized exercises into (dynamic) resistance exercise (DRT), weight bearing (WB) exercise and combined WB&DRT exercise. Our hypotheses were that all types of exercise significantly affect BMD at (1) LS, (2) FN and (3) total hip (TH) (4), albeit without significant differences between the exercise categorizations at any BMD-ROI.

## Material and Methods

The present study is based on a comprehensive systematic review of the effect of exercise on (areal) BMD in postmenopausal women [3] to which the reader is kindly referred for details.

### Data Sources and Search Strategy

We strictly followed the Preferred Reporting Items for Systematic Reviews and Meta-Analyses (PRISMA) statement [10]; and fully registered the study in PROSPERO (CRD42018095097). Briefly eight databases (PubMed, Scopus, Web of Science, Cochrane, Science Direct, Eric, ProQuest and Primo) were searched for articles published up to March 1, 2019 without language restrictions.

The search strategy comprised a combination of population, intervention, and outcomes. Databases were systematically searched around the following combination of terms: “Bone Mineral Density”, “Exercise”, and “Postmenopausal”. Following the primary search and duplicate exclusion, the same reviewer (MS) screened studies by title and abstracts according to the eligibility criteria. A manual search in the reference lists of all included articles was carried out in an attempt to find new relevant studies. Authors of trials that were potentially eligible were contacted by e-mail for any missing data (e.g., mean change of BMD or SD) or clarification of data presented.

### Inclusion and Exclusion Criteria

We included studies/study arms with (1) randomized and non-randomized controlled protocols with at least one exercise group versus one control group with sedentary/habitual active lifestyle or placebo exercise; (2) women who were postmenopausal at study start; (3) ≥ 6 months intervention duration; (4) areal BMD of the LS, femoral neck (FN) and/or total hip (tH) region at baseline and follow-up assessment as determined by (5) dual-energy X-ray absorptiometry (DXA) or dual-photon absorptiometry (DPA); (6) ≤ 10% of women on osteoanabolic/antiresorptive, or osteocatabolic (glucocorticoids) pharmaceutic agents; albeit only when the number of subjects was comparable between exercise and control.

We further excluded studies with (1) mixed gender or mixed pre- and postmenopausal cohorts without separate BMD analyses; (2) women undergoing chemo- and/or radiotherapy and (3) women with diseases that relevantly affect bone metabolism. (4) Duplicates from one study and (5) review articles, case reports, editorials, conference abstracts, and letters were not considered. Lastly, exercise study groups (see below) that cannot be classified on the intended type of exercise were also excluded from the present analysis.

### Data Extraction

We designed a pre-piloted extraction form to extract relevant data. The form asked for details with respect to publication characteristics, methodology, participant characteristics, exercise characteristics, risk assessment and outcome characteristics. Two reviewers (SvS and MS) independently evaluated full-text articles and extracted data from the included studies, in case of inconsistency, a third reviewer decided (WK).

### Outcome Measures

The primary outcome was change of (areal) BMD at LS-, FN- and TH-ROI as assessed by DXA or DPA between baseline and follow-up. In cases of multiple BMD assessments, we considered only changes between the baseline and final BMD assessments.

### Quality Assessment

All studies included were independently assessed for risk of bias by two independent raters (WK and MV) using the Physiotherapy Evidence Database (PEDro) scale [11]. In case of inconsistency, a third reviewer decided (SvS).

### Data Synthesis

For the detailed procedure to impute missing standard deviations (SD) the reader is kindly referred to the comprehensive meta-analysis of Shojaa et al. [3]. Briefly, if the studies presented a confidence interval (CI) or standard errors (SE), they were converted to SD. In cases of missing CI or SE data we first contacted authors (*n* = 11) to provide corresponding information. When no reply was received or data were not available, the exact p-value of the absolute change of BMD was obtained to compute the SD of the change. In the case of unreported p-value, we calculated the SDs using pre and post SDs.

In order to determine the effects of different types of exercise we categorized the studies according to the following approach: (a) dynamic resistance exercise, i.e., any kind of resistance exercise that involves joint movement to develop musculoskeletal strength. We focus on studies that applied isolated DRT without any adjuvant exercise component and without bone-specific warm ups (e.g., running, hopping, aerobic dance) with validated effect on bone [1, 4, 5], (b) weight bearing exercise that involved any kind of aerobic and anaerobic loading of axial skeletal sites due to gravity, i.e., Tai Chi, walking, running, dancing, movement games, heel drops, hopping, jumping and (c) exercise studies that combined weight bearing and DRT exercise, even if WB exercise was applied only shortly during warm up. The latter approach was selected due to the observation that only few cycles with high strain rates may induce positive effects on bone [12, 13]. Two raters (WK and MV) independently categorized the data, in case of inconsistency, a third reviewer decided (SvS).

### Statistical Analysis

The statistical analysis was performed using the statistical software R (R Development Core Team) [14]. Effect size (ES) value was considered as the standardized mean differences (SMDs) combined with the 95% confidence interval (95% CI). Random-effects meta-analysis was performed by applying the metafor package [15]. Heterogeneity for between-study variability was determined using the Cochran *Q* test, as with other statistical analyses a *P*-value < 0.05 was considered significant. The level of heterogeneity was analyzed with the *I*^2^ statistic. For those studies with two different intervention groups, the control group was proportionally split into two groups for comparison against each intervention group [16]. Sensitivity analysis was conducted to check whether the overall result of the analysis is robust regarding the use of the imputed SDs. Funnel plots with regression test and the rank correlation between effect estimates and their standard errors, using the t-test and Kendall’s τ statistic respectively, were applied to explore potential publication bias. To adjust the results for possible publication bias, we also conducted a trim and fill analysis using the L0 estimator proposed by Duval et al. [17]. The present subgroup analysis was conducted as a mixed-effect meta-analysis with “type of exercise” as the moderator. A *P*-value of < 0.05 was considered as significant for all tests.

## Results

### Study Characteristics

**Fig. 1:**
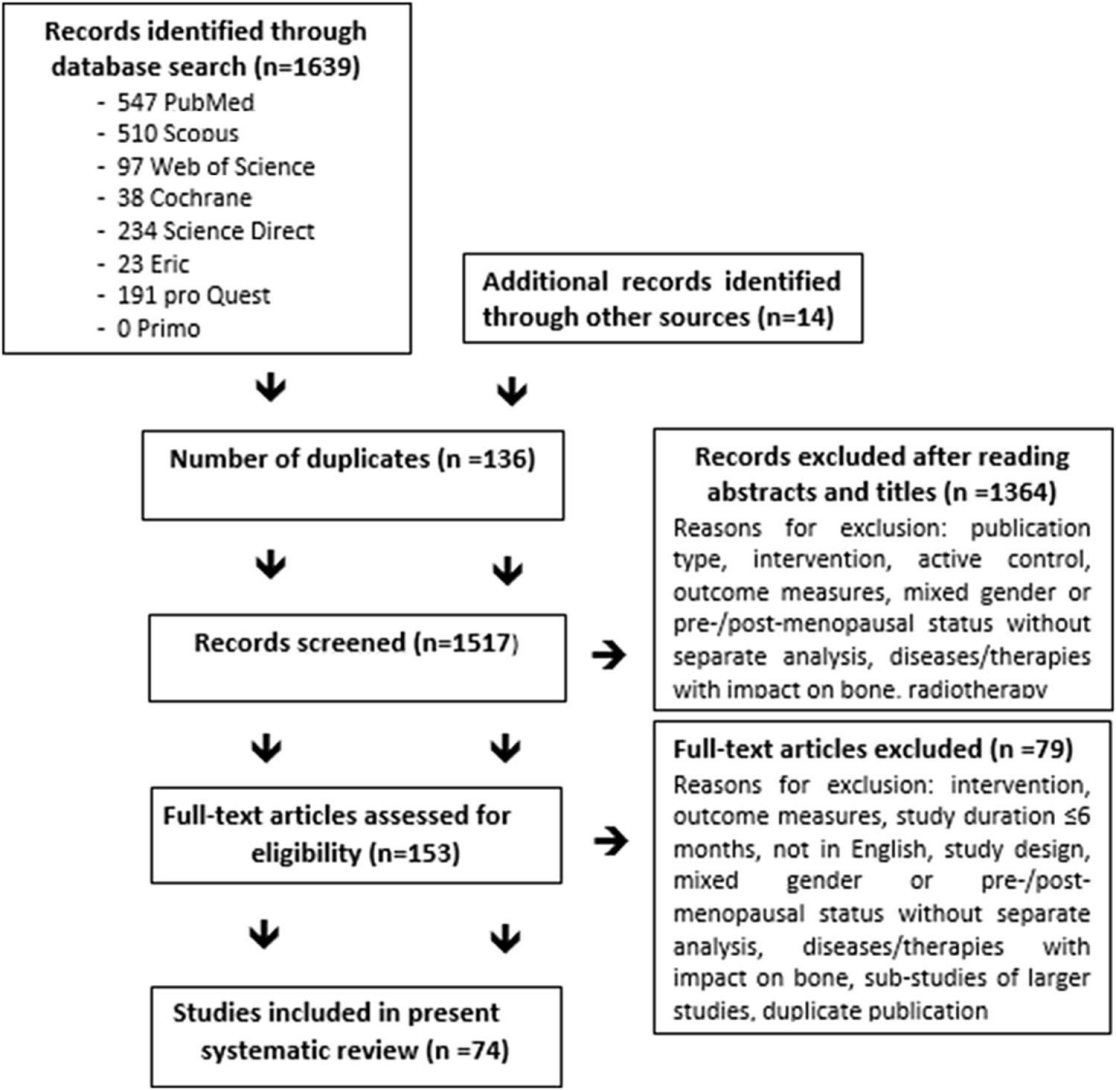
Flow diagram of search process according to PRISMA [10]

In total, our search identified 74 eligible studies with 84 exercise groups (Fig. 1), categorized into 18 DRT groups [18–32], 30 weight bearing (WB) type exercise groups [31–57] and 36 study groups that scheduled combined exercise protocols [33, 48, 58–88].

The pooled number of participants was 2793 in the exercise and 2319 in the control group respectively. In detail, the number of participants in exercise and control was 1344 and 1175 women in the combined WB DRT group, 1045 and 815 women in the WB and 404 and 329 women in the DRT group. Table 1 gives the anthropometric participant characteristics of the included studies.

**Table 1:** Participants characteristics of included studies

First author, year	Sample size (*n*)	Age (years)	Menopausal age (years)	Body mass (kg)	Height (cm)	BMI (kg/m^2^)
Adami, 1999	E: 125 C: 125	E: 65 ± 6 C: 63 ± 7	E: 16 ± 7 C: 14 ± 8	n.g n.g	n.g n.g	E: 24.6 ± 3.3 C: 23.8 ± 3.8
Basat, 2013	RE: 14 HI: 14 C: 14	RE: 56 ± 5 HI: 56 ± 3 C: 56 ± 4	RE: 6 ± 4 HI: 7 ± 2 C: 6 ± 3	n.g n.g n.g	n.g n.g n.g	RE: 25 ± 4.7 HI: 26.4 ± 3.5 C: 27.5 ± 3.7
Bassey, 1995	E: 31^a^ C: 32	E: 54 ± 4 C: 55 ± 3	E: 7 ± 4 C: 7 ± 5	E: 63.3 ± 11.4 C: 64.7 ± 6.7	E: 163 ± 6 C: 159 ± 5	E: 24.6 ± 2.7 C: 24.9 ± 3.8
Bassey, 1998	E: 45 C: 32	E: 56 ± 3 C: 55 ± 4	E: 7 ± 4 C: 5 ± 4	E: 64.7 ± 7.3 C: 66.5 ± 7.8	E: 161 ± 6 C: 163 ± 6	E: 25 ± 2.6 C: 25.1 ± 2.6
Bello, 2014	E: 10 C: 10	E: 61 ± 6 C: 61 ± 6	n.g n.g	n.g n.g	n.g n.g	n.g n.g
Bemben, 2010	E: 22^b^	E: 64 ± 1		E: 76.6 ± 3.2	E: 161 ± 2	E: 30 ± 1
	C: 12	C: 63 ± 1	> 5	C: 77.9 ± 4.5	C: 163 ± 1	C: 29 ± 1
Bemben, 2000	HR: 11 HL: 13 C: 11	HL: 50 ± 2 HR: 52 ± 2 C: 52 ± 1	HL: 4 ± 1 HR: 2 ± 1 C: 3 ± 1	HL: 74.7 ± 5.6 HR: 62.7 ± 3.4 C: 66.5 ± 4.2	HL: 162 ± 2 HR: 165 ± 2 C: 166 ± 2	HL: 28.7 ± 2.4 HR: 23.2 ± 1.2 C: 24.2 ± 1.7
Bergström, 2008	E: 60 C: 52	E: 59 ± 4 C: 60 ± 3	n.g n.g	n.g n.g	n.g n.g	E: 24.4 ± 2.6 C: 24.9 ± 2.3
Bocalini, 2009	E: 23 C: 12	E: 69 ± 9 C: 67 ± 8	n.g n.g	E: 68 ± 6 C: 69 ± 7	n.g n.g	E: 28 ± 4 C: 27 ± 6
Bolton, 2012	E: 19 C: 20	E: 60 ± 6 C: 56 ± 5	E: 13 ± 7 C: 12 ± 7	E: 64.5 ± 9.7 C: 63.6 ± 11.9	E: 160 ± 4 C: 160 ± 6	E: 25.2 ± 4.3 C: 25 ± 4.4
Brooke-Wavell, 1997	E: 43 C: 41	E: 65 ± 3 C: 64 ± 3	E: 15 ± 5 C: 15 ± 7	E: 67.7 ± 10.9 C: 67.9 ± 10.6	E: 162 ± 6 C: 163 ± 7	E: 25.8 ± 3.8 C: 25.6 ± 3.5
Brooke-Wavell, 2001	E: 18 C: 21	E: 65 ± 3 C: 65 ± 3	> 5	E: 68.5 ± 8.9 C: 71.4 ± 12.1	E: 163 ± 7 C: 164 ± 7	n.g n.g
Caplan, 1993^h^	E: 19 C: 11	E: 66 ± 1 C: 65 ± 1	E: 18 ± 2 C: 21 ± 3	E: 63.2 ± 2.5 C: 60.6 ± 2.9	E: 158 ± 2 C: 160 ± 2	E: 25.4 ± 0.9 C: 23.5 ± 0.8
Chan, 2004	E: 67 C: 65	E: 54 ± 3 C: 54 ± 3	E: 5 ± 2 C: 4 ± 2	E: 55.4 ± 7.9 C: 54 ± 10.3	E: 150 ± 10 C: 150 ± 20	E: 24.1 ± 4.7 C: 23.5 ± 4.6
Chilibeck, 2013	E + Pl: 86 Pl: 88	E + Pl: 55 ± 6 Pl: 56 ± 7	> 1	E + Pl: 73.4 ± 14.1 Pl: 73.6 ± 15.9	E + Pl: 163 ± 5 Pl: 163 ± 6	n.g n.g
Chilibeck, 2002^h^	E: 14 C: 14	E: 57 ± 2 C: 59 ± 2	E: 9 ± 2 C: 8 ± 2	E: 72 ± 4.3 C: 73.2 ± 4.8	E: 164 ± 2 C: 165 ± 1	E: 27 ± 1.7 C: 26.6 ± 1.2
Choquette, 2011	E + Pl: 25 Pl: 26	E + Pl: 58 ± 6 Pl: 59 ± 6	E + Pl: 8 ± 8 Pl: 10 ± 8	E + Pl: 75.4 ± 12.1 Pl: 79.5 ± 9.2	E + Pl: 161 ± 6 Pl: 160 ± 6	E + Pl: 29.1 ± 3.9 Pl: 31 ± 2.9
Chuin, 2009	E + Pl: 11 Pl: 7	E + Pl: 65 ± 3 Pl: 67 ± 4	n.g n.g	E + Pl: 66.6 ± 8.5 Pl: 64.2 ± 7.6	n.g n.g	E + Pl: 26.5 ± 2.7 Pl: 26 ± 2.8
de Matos, 2009	E: 30 C: 29	E: 57 ± 5 C: 57 ± 5	10 7	E: 59.8 ± 7.6 C: 65 ± 8.3	E: 158 ± 4 C: 159 ± 8	E: 23.9 ± 3.3 C: 25.6 ± 3.1
Deng, 2009	E: 45 C: 36	E: 54 ± 4 C: 51 ± 5	E: 4 ± 3 C: 3 ± 2	E: 58.8 ± 8 C: 58.3 ± 7.5	E: 157 ± 5 C: 159 ± 5	n.g n.g
de Oliveira, 2018	E: 17 C: 17	E: 56 ± 7 C: 54 ± 5	E: 8 ± 7 C: 9 ± 7	E: 67.4 ± 8.6 C: 64.6 ± 6.6	E: 157 ± 6 C: 154 ± 4	E: 27.2 ± 2.7 C: 27.3 ± 2.5
Duff, 2016	E: 22 C: 22	E: 65 ± 5 C: 65 ± 5	n.g n.g	n.g n.g	E: 162 ± 6 C: 160 ± 7	n.g n.g
Ebrahim, 1997	E: 81 C: 84	E: 66 ± 8 C: 68 ± 8	n.g n.g	n.g n.g	n.g n.g	E: 26.6 ± 4.3 C: 26.3 ± 4.8
Englund, 2005	E: 24 C: 24	E: 73 ± 4 C: 73 ± 5	n.g n.g	E: 66.9 ± 8.7 C: 67.7 ± 8.5	E: 162 ± 6 C: 160 ± 6	E: 25.2 ± 2.7 C: 26.1 ± 3.2
Evans, 2007	E + SP: 11^c^ SP: 10	E + SP: 62 ± 5 SP: 63 ± 5	E + SP: 8 ± 6 SP: 8 ± 5	E + SP: 66.7 ± 13.3 SP: 67.6 ± 7.3	E + SP: 163 ± 7 SP: 161 ± 6	n.g n.g
Going, 2003	E: 91 C: 70	E: 56 ± 5 C: 57 ± 5	> 3	E: 68.9 ± 11.4 C: 67.8 ± 11.4	E: 163 ± 7 C: 163 ± 5	E: 25.8 ± 3.4 C: 25.5 ± 4
Grove, 1992	LI: 5 HI: 5 C: 5	LI: 57 ± 4 HI: 54 ± 2 C: 56 ± 4	LI: 3 ± 2 HI: 4 ± 3 C: 4	LI: 69 ± 12.7 HI: 72.3 ± 19.2 C: 70.5 ± 10.1	n.g n.g n.g	n.g n.g n.g
Hans, 2002	E: 110 C: 35	E: 68 ± 5 C: 66 ± 5	> 5	E: 63 ± 7.3 C: 59.5 ± 7.5	E: 161 ± 8 C: 159 ± 8	n.g n.g
Hartard, 1996	E: 18 C: 16	E: 64 ± 6 C: 67 ± 10	> 2	E: 67 ± 7.7 C: 63.8 ± 11.2	E: 162 ± 7 C: 158 ± 6	n.g n.g
Hatori, 1993	E–H: 12^d^ E-M: 9 C: 12	H: 56 ± 4 M: 58 ± 5 C: 58 ± 8	H: 7 ± 5 M: 6 ± 4 C: 9 ± 8	H: 54 ± 5 M: 53.4 ± 6.8 C: 53.9 ± 6	H: 151 ± 3 M: 151 ± 5 C: 151 ± 5	H: 23.3 ± 2.3 M: 23.5 ± 2.4 C: 24.6 ± 3.3
Iwamoto, 2001	E: 8 C: 20	E: 65 ± 5 C: 65 ± 6	E: 16 ± 6 C: 15 ± 6	E: 45.5 ± 6.5 C: 45.8 ± 4	E: 152 ± 8 C: 152 ± 6	E: 19.7 ± 1.3 C: 19.9 ± 2.1
Jessup, 2003	E: 10 C: 10	E: 69 ± 3 C: 69 ± 4	E: 24 ± 11 C: 22 ± 11	E: 78 ± 9.2 C: 84.2 ± 17.7	n.g n.g	n.g n.g
Karakiriou, 2011^h^	E: 10 C: 9	E: 53 ± 1 C: 53 ± 1	E: 5 ± 1 C: 3 ± 1	E: 71.2 ± 2.8 C: 75.4 ± 2	E:159 ± 1 C:157 ± 2	E: 28.1 ± 1.1 C: 30.4 ± 0.8
Kemmler, 1999	E-PM: 15 L-PM: 17 C: 18	E-PM: 54 ± 5 L-PM:65 ± 6 C: 56 ± 8	E-PM ≤ 8 L-PM > 8 C > 1	n.g n.g n.g	n.g n.g n.g	E-PM: 25.5 ± 4.2 L-PM: 26.2 ± 3.8 C: 27.4 ± 5.3
Kemmler, 2004	E: 86 C: 51	E: 55 ± 3 C: 56 ± 3	> 1	E: 67.6 ± 9.7 C: 64.8 ± 13.6	E: 164 ± 6 C: 162 ± 7	E: 25.1 ± 3.3 C: 24.7 ± 3.9
Kemmler, 2010	E: 123 C: 123	E: 69 ± 4 C: 69 ± 4	n.g n.g	E: 68.1 ± 10.9 C: 69.5 ± 12	E: 162 ± 6 C: 160 ± 6	n.g n.g
Kemmler, 2013	E: 43 C: 42	E: 52 ± 2 C: 52 ± 3	E: 2 ± 1 C: 2 ± 1	E: 69.5 ± 9.6 C: 70.9 ± 16.8	E: 165 ± 5 C: 165 ± 6	n.g n.g
Kerr, 2001	RE: 42 Fit: 42 C: 42	RE: 60 ± 5 Fit: 59 ± 5 C: 62 ± 6	RE: 11 ± 6 Fit: 9 ± 5 C: 12 ± 6	RE: 72.2 ± 12 Fit: 69 ± 11.4 C: 69.3 ± 14.6	RE: 163 ± 5 Fit: 165 ± 6 C: 162 ± 7	n.g n.g n.g
Kerr, 1996	En: 28^e^ S: 28	En: 56 ± 5 S: 58 ± 4	En: 6 ± 4 S: 8 ± 3	En: 70.8 ± 10 S: 69.4 ± 11.4	En: 165 ± 6 S: 165 ± 7	n.g n.g
Kohrt, 1997^h^	JRF: 15 GRF: 18 C: 15	JRF: 65 ± 1 GRF: 66 ± 1 C: 68 ± 1	n.g n.g n.g	JRF: 72.6 ± 2.3 GRF: 70.9 ± 4.2 C: 71.6 ± 1.8	JRF: 164 ± 2 GRF: 163 ± 1 C: 163 ± 2	n.g n.g n.g
Kohrt, 1995	E: 8^f^ C: 8	E: 65 ± 3 C: 66 ± 3	> 10	E: 63.4 ± 11.9 C: 63.4 ± 8.1	E: 161 ± 5 C: 161 ± 5	n.g n.g
Korpelainen, 2006	E: 84 C: 76	E: 73 ± 1 C: 73 ± 1	n.g n.g	E: 61.2 ± 7.9 C: 62.2 ± 9.2	E: 154 ± 5 C: 156 ± 5	E: 25.7 ± 3.4 C: 25.5 ± 3.5
Kwon, 2008	E: 20 C: 20	E: 77 ± 2 C: 77 ± 3	n.g n.g	E: 56.4 ± 3.8 C: 58.1 ± 5.6	E: 149 ± 6 C: 152 ± 3	E: 25.9 ± 1.9 C: 25.2 ± 2.8
Lau, 1992	E + Pl: 15 Pl: 15	E + Pl: 79 Pl: 75	n.g n.g	n.g n.g	n.g n.g	n.g n.g
Liu, 2015	E: 50 C: 48	E: 63 ± 7 C: 62 ± 8	E: 14 ± 6 C: 13 ± 7	n.g n.g	E: 154 ± 4 C: 157 ± 4	n.g n.g
Lord, 1996	E: 90 C: 89	E: 72 ± 5 C: 71 ± 5	n.g n.g	E: 66 ± 11.4 C: 64.7 ± 14.4	E: 157 ± 6 C:157 ± 7	n.g n.g
Maddalozzo, 2007	E: 35 C: 34	E: 52 ± 3 C: 52 ± 3	E: 2 ± 1 C: 2 ± 1	E: 70 ± 8.7 C: 67.1 ± 12.6	n.g n.g	n.g n.g
Marques, 2011	E: 30 C: 30	E: 70 ± 5 C: 68 ± 5	n.g n.g	n.g n.g	n.g n.g	E: 28.4 ± 3.7 C: 28.2 ± 3.7
Marques-Wanderley, 2011	RE: 23 AE: 24 C: 24	RE: 67 ± 5 AE: 70 ± 5 C: 68 ± 6	n.g n.g n.g	n.g n.g n.g	n.g n.g n.g	RE: 28.8 ± 4.6 AE: 27.5 ± 3.8 C: 28.1 ± 3.5
Martin, 1993	45^min^E:25 30^min^E:27 C: 24	45^ min^: 58 ± 7 30^ min^: 60 ± 8 C: 57 ± 7	45^ min^: 9 ± 9 30^ min^: 13 ± 9 C: 8 ± 7	45^ min^: 65.6 ± 11.9 30^ min^: 68.9 ± 11.5 C: 72.9 ± 15.5	45^ min^: 159 ± 5 30^ min^: 162 ± 7 C: 162 ± 4	n.g n.g n.g
Milliken, 2003	E: 26 C: 30	E: 57 ± 5 C: 57 ± 5	E: 6 ± 3 C: 6 ± 3	E: 68.4 ± 10.6 C: 68.4 ± 10.6	E: 162 ± 6 C: 162 ± 6	n.g n.g
Nelson, 1994	E: 21 C: 19	E: 61 ± 4 C: 57 ± 6	E: 12 ± 5 C: 10 ± 5	E: 64.7 ± 7.7 C: 62.2 ± 8.9	E: 163 ± 6 C: 164 ± 8	E: 24.4 ± 2.5 C: 23.1 ± 2.2
Nelson, 1991^h^	E: 21^g^ C: 20	E: 60 ± 1 C: 60 ± 1	E: 11 ± 1 C: 11 ± 1	E: 64 ± 1.4 C: 64 ± 1.4	E: 162 ± 1 C: 162 ± 1	E: 24.4 ± 0.5 E: 24.4 ± 0.5
Nichols, 1995^h^	E: 17 C: 17	E: 68 ± 2 C: 65 ± 1	E: 18 ± 1 C: 18 ± 1	E: 68.8 ± 2.8 C: 72 ± 13.5	E: 163 ± 1 C: 164 ± 1	n.g n.g
Nicholson, 2015	E: 28 C: 29	E: 66 ± 4 C: 66 ± 5	> 5	E: 70.6 ± 9.1 C: 66.8 ± 10.7	E: 164 ± 4 C: 163 ± 5	E: 26 ± 3.2 C: 24.5 ± 2.9
Orsatti, 2013	E + Pl: 20 Pl: 20	E + Pl: 56 ± 9 Pl: 55 ± 8	E + Pl: 9 ± 6 Pl: 8 ± 6	n.g n.g	n.g n.g	E + Pl: 26 ± 3 Pl: 30.4 ± 5.3
Park, 2008	E: 25 C: 25	E: 68 ± 4 C: 68 ± 3	E: 18 ± 2 C: 19 ± 3	n.g n.g	E: 153 ± 4 C: 152 ± 4	n.g n.g
Prince, 1995	E + Ca: 42 Ca: 42	E + Ca: 63 ± 5 Ca: 62 ± 5	E + Ca: 16 ± 5 Ca: 16 ± 6	n.g n.g	n.g n.g	n.g n.g
Pruitt, 1992^h^	E: 17 C: 10	E: 54 ± 1 C: 56 ± 1	E: 3 C: 4 ± 1	E: 64.2 ± 1.9 C: 65.5 ± 2.9	E: 162 ± 1 C: 163 ± 2	n.g n.g
Pruitt, 1995	H-int: 15 L-int: 13 C: 12	H-int: 67 ± 1 L-int: 68 ± 1 C: 70 ± 4	n.g n.g n.g	H-int: 64.5 ± 9.2 L-int: 61.5 ± 4.6 C: 63.8 ± 9.1	H-int: 162 ± 7 L-int: 160 ± 5 C: 160 ± 9	H-int: 24.5 ± 3.4 L-int: 23.9 ± 1.6 C: 25.1 ± 3.1
Ryan, 1998	E: 18 C: 18	E: 62 ± 6 C: 63 ± 6	> 2	E: 79.3 ± 8 C: 83.1 ± 11.3	n.g n.g	E: 30.5 ± 2.8 C: 30.9 ± 3
Sakai, 2010^h^	E: 49 C: 45	E: 68 ± 1 C: 68	n.g n.g	E: 51.4 ± 1.1 C: 51.7 ± 0.9	E: 151 ± 1 C: 151 ± 1	E: 22.4 ± 0.4 C: 22.6 ± 0.4
Silverman, 2009	E: 46 C: 40	E: 60 ± 5 C: 58 ± 5	E: 12 ± 8 C: 11 ± 7	E: 84.6 ± 11.3 C: 87.4 ± 14.4	n.g n.g	E: 32.1 ± 4.2 C: 32.6 ± 4.6
Sinaki, 1989	E: 34 C: 34	E: 56 ± 4 C: 56 ± 4	> 0.5	E: 66.2 ± 9.3 C: 66.1 ± 10.6	E: 163 ± 6 C: 161 ± 5	n.g n.g
Sugiyama, 2002^h^	E: 13 C: 13	E: 52 ± 1 C: 53 ± 1	E: 3 C: 2	E: 54.7 ± 3.4 C: 50.9 ± 1.7	E: 155 ± 2 C: 153 ± 1	E: 22.7 ± 1.2 C: 21.7 ± 0.7
Tartibian, 2011	E: 20 C: 18	E: 61 ± 7 C: 59 ± 8	> 8	E: 77.5 ± 10.4 C: 75.9 ± 17.2	E: 167 ± 8 C: 168 ± 16	E: 25.1 ± 7.1 C: 28.5 ± 3.7
Tolomio, 2009	E: 81 C: 79	E: 62 ± 5 C: 64 ± 5	n.g n.g	E: 66 ± 10.9 C: 63 ± 9.7	E: 161 ± 10 C: 159 ± 10	n.g n.g
Verschueren, 2004	E: 22 C: 24	E: 64 ± 4 C: 64 ± 3	E: 15 ± 6 C: 15 ± 7	E: 70.5 ± 9.6 C: 68.6 ± 14.5	E: 161 ± 6 C: 160 ± 6	E: 27.4 ± 3.5 C: 26.5 ± 5.8
Wang, 2015	TC: 40 TC + RT:40 C: 39	TC: 58 ± 3 TCRT: 58 ± 3 C: 58 ± 3	> 0.5	TC: 60.5 ± 8.3 TCRT: 60 ± 6 C: 60.5 ± 8.3	TC: 159 ± 5 TCRT: 161 ± 4 C: 159 ± 5	n.g n.g n.g
Woo, 2007	TC: 30 RE: 30 C: 30	TC: 70 ± 3 RE: 70 ± 3 C: 69 ± 3	n.g n.g n.g	n.g n.g n.g	n.g n.g n.g	TC: 24.4 ± 4.3 RE: 24.6 ± 4 C: 24.9 ± 3
Wu, 2006	E + Pl: 34 Pl: 34	E + Pl: 55 ± 3 Pl: 55 ± 3	E + Pl: 4 ± 2 Pl: 4 ± 2	E + Pl: 54.1 ± 7.3 Pl: 51.4 ± 7.1	E + Pl: 155 ± 6 Pl: 157 ± 6	E + Pl: 22.4 ± 2.9 Pl: 20.9 ± 2.2
Yamazaki, 2004^ h^	E: 32 C: 18	E: 64 ± 3 C: 66 ± 3	E: 17 ± 2 C: 15 ± 2	E: 51.2 ± 1.4 C: 50.1 ± 1.6	E: 155 ± 1 C: 156 ± 1	E: 21.2 ± 0.7 C: 21.1 ± 1.1

All values are presented as mean ± SD, if not otherwise stated

*AE* aerobic exercise, *C* control, *Ca* calcium, *E* exercise, *En* Endurance, *E-PM* early postmenopausal, *Fit* fitness, *GRF* ground reaction forces (i.e., walking), *H* high, *HI* high impact, *H-int* high intensity, *HL* high load, *HR* high repetition, *JRF* joint-reaction forces, *LI* low impact, *L-int* Low intensity, *L-PM* late postmenopausal, *M* Moderate, *n.g.* not given, *Pl* placebo, *RE* resistance exercise, *S* strength, *SP* soy protein, *TCRT* Tai Chi resistance training, *TC* Tai Chi

^a^63 women were randomized equally

^b^Not stated to which groups the 7 drop out belong

^c^Not stated to which groups the 9 drop outs belong

^d^Not clear to which groups the two persons who failed to complete the program belong

^e^Unilateral exercise: One side of body as control and the other side as intervention

^f^No data concerning participants/group; we assumed an equal allocation

^g^Exercise with or without Ca vs sedentary control with or without Ca-supplements

^h^Numbers are presented as mean ± SE

Sample sizes in the exercise arms ranged from 5 [28] to 125 participants [58] per group (CG: 2 to 125 women). Thirteen studies [22, 33, 41, 45, 47, 61, 63, 68, 73, 75, 77, 87, 89] included women with osteopenia/osteoporosis (DRT: *n* = 1 vs. WB: *n* = 4 vs. WB&DRT: *n* = 8); (Table 2). Average age varied among the studies between 51 ± 2 years [18] and 77 ± 3 years [81]. Twelve studies with fourteen exercise groups [18, 24, 28, 38, 55, 57, 69, 72, 75] focused on cohorts of “early postmenopausal women” (1 to ≤ 8 years post). Thirty studies included participants with sedentary/habitually active lifestyles, 28 trials involved participants with exercise activities presumably with minor effects on bone and 16 studies did not provide the corresponding information (Table 2).

**Table 2:** Exercise prescription characteristics of included studies, categorized according the type of exercise

First author, year	Health-, menopausal- and exercise -status	Duration (months)	Progression intensity	Types and specifications of the exercise content	Site specificity	Volume (min/week), supervision (attendance)	Exercise/strain composition and further details of the protocol
*WB type exercise (n = 30)*							
Basat, 2013	Osteopenia 6 ± 4 y post No-BSE	6	No	Rope skipping	? Yes	7 × 35, S-JE (> 60%)	15 min warm up (walking), maximum 50 jumps/session (more details n.g.)
Bassey, 1995	Healthy 7 ± 4 y post No-BSE	12	No	Heel drops, jumping, skipping	? Yes	1 × ?, S-JE 7 × ?, HE (84%)	HE: 50 heel drops barefoot on a thinly covered floor with knee & hip extended. S-JE: jumping & skipping (more details n.g.)
Bassey, 1998	Healthy 7 ± 4 y post No vigorous Ex > 1 h/w	12	No	Jumping: counter-movement jumps (CMJ)	? Yes	5 × 10, HE 1 × 10, S-JE (91%)	50 CMJ barefoot with both legs, 5 sets × 10 reps with ground reaction forces (GRF): 4 × body mass
Brooke-Wavell, 2001	Healthy > 5 y post Sedentary	12	No	Brisk walking	No Yes	> 3x > 20 (140 min/w), Non-supervised (> 90%)	4–5 × 25–35- min/d ≈ 70% HRmax
Brooke-Wavell, 1997	Healthy 15 ± 6 y post Sedentary	12	No	Brisk walking	No Yes	140 min/w, Non-supervised (100%)	20–50 min long for each walk, ≈ 70% HRmax
Chan, 2004	Healthy 5 ± 2 y post No > 0.5 h/w	12	No	Tai Chi: Yang-Style (all main muscle groups (more details n.g.))	? Yes	5 × 50, S-JE (≈ 84%)	Slow, smooth movements with constant velocity
Ebrahim, 1997	Healthy (upper limb fractures) 66 ± 8 y No limit	24	No	Brisk walking	No Yes	3 × 40, HE (100%)	40 min walking, “faster than usual, but not so fast as to be uncomfortable “
Evans, 2007	Healthy ≈ 8 ± 6 y post n.g	9	Yes	Walking/running, stairclimbing (machines)	? Yes	3 × 45, S-JE (n.g.)	WB & Non-WB AET (machines) at 55–80% VO_2_peak. Rest by changing exercise mode
Hans, 2002	≥ Osteopenia > 5 y post n.g	24	Yes (?)	Heel drops: barefoot on a force measuring platform (osteocare)	? Yes	5 × 3–5, HE (65%)	Impact loading: strength or height 25–50% above the estimated resting force, daily 120 correct force impacts
Hatori, 1993	Healthy ≈ 7 ± 5 y post n.g	7	No	Walking below the anaerobic threshold	No Yes	3 × 30, n.g (n.g.)	30 min walking at 90% anaerobic threshold HR (6.2 km/h) at “flat grass covered ground”
		7	No	Walking above the anaerobic threshold	No Yes	3 × 30, n.g (n.g.)	30 min walking at 110% anaerobic threshold HR (7.2 km/h) at “flat grass covered ground”
Kohrt, 1995	Healthy > 8 y post Sedentary	11	Yes	Walking, jogging, stair climbing	? Yes	3–5 × 45, HE (≈ 70%)	First 2 months flexibility, 9 months WB: 5–10 min warm up (treadmill 60–70% HRmax), 30 min WB at 65–85% HRmax
Kohrt, 1997	Healthy > 8 y post Sedentary	11	Yes	Walking, jogging, stair climbing	? Yes	3–5 × 30–45, n.g. (presumably S-HE) (≈ 70%)	First 2 months flexibility, 9 months WB at 60–85% HRmax
Korpelainen, 2006	Osteopenia> 8 y post n.g	30	Yes	Jumping, walking/jogging, dancing, stamping, chair climbing	? Yes	1 × 60, S-JE 7 × 20, HE (≈ 75%)	S-JE: 45 min WB AET. The first six months: 1 × 60 min S-JE & daily × 20 min HE. The second six months: HE: daily × 20 min HE applying the same exercise to S-JE
Lau, 1992	Healthy > 8 y post n.g	10	No	Stepping up and down, Upper trunk movements	? Yes	4× ≈ 20–25, S-JE (n.g.)	100 steps on a 23 cm block 15 min upper trunk movements (?) in a standing position with sub-maximum effort (more details n.g.)
Liu, 2015	Osteoporosis 14 ± 6 y post n.g	12	No	Tai Chi	? Yes	3 × daily ≈ 3–5,HE (96%)	8 exercise brocade, 7 reps (slowly raising the arms, moving to tip toe, stretching the back and go back on the heel with arms hanging down)
Marques, 2011	Healthy > 8 y post Sedentary	8	Yes	Walking, stepping, skipping, jogging, dancing, DRT (first 6 weeks only)	? Yes	3 × 60, S-JE (78%)	Only the first 6 w 10 min DRT (lower body). 35–40 min of WB AET (50–85% HRR) with Peak-GRF up to 2.7 × body mass with up to 120 beats/min
Martin, 1993	Healthy ≈11 ± 9 y post No-BSA	12	Yes	Brisk walking on treadmill	No Yes	3 × 36–40, n.g. (presumably S-JE) (79%)	30 min brisk walking (4–6.2 km/h at 3–7% incline) at 70–85% HRmax
		12	Yes	Brisk walking on treadmill	No Yes	3 × 51–55, n.g. (presumably S-JE) (82%)	45 min brisk walking (4–6.2 km/h at 3–7% incline) at 70–85% HRmax
Nelson, 1991	Healthy 11 ± 1 y post Sedentary	12	No	Walking with weighted vest	No Yes	4 × 50, S-JE (90%)	Walking with a 3.1 kg weighted vest at 75–80% HRmax
Prince, 1995	Healthy > 8 y post ≤ 2 h/w Ex	24	No	WB AET (more details n.g.)	? Yes	4 × 60, 2 × S-JE/2 × HE (39%)	4 × WB exercise (including 2 × walking) at 60% HRmax (more details n.g.)
Ryan, 1998	Healthy > 2 y post Sedentary	6	Yes	Walking, jogging on treadmill	? Yes	3 × 55, S-E (> 90%)	Up to (4th month) 35 min walking/jogging at 50–70% VO_2_max, 10 min cool down (cycle ergometer), Energy-intake restriction of 250–350 kcal/d (weight loss study)
Sakai, 2010	Healthy > 8 y post n.g	6	No	Unilateral standing on one leg	No Yes	7 × 2, HE (≥ 70%)	3 sets (early, at noon, in the evening) of unilateral standing for 1 min on each leg with eyes open
Silverman, 2009	Healthy 12 ± 8 y post Sedentary	6	No	Walking	No Yes	3 × 45–60, S-JE > 1session (78%)	walking at 50–75% HRmax, energy-intake restriction of 250–350 kcal/d (weight loss study)
Sugiyama, 2002	Healthy 3 y post n.g	6	No	Rope skipping (more details n.g.)	? Yes	2–3 × ?, HE (82%)	100 jump/session (more details n.g.)
Tartibian, 2011	Healthy > 8 y post Sedentary	6	Yes	Walking/jogging on treadmill	? Yes	3–6 × 25–45, S-JE (95%)	First 12 weeks: 3–4 × 25–30 min at 45–55% HRmax, second 12 weeks: 4–6 × 40–45 min at 55–65% HRmax
Wang, 2015	Healthy > 0.5 y post No Tai Chi	12	No	Tai Chi (Yang-style)	? Yes	2 × 60, S-JE 2 × 60, Group E with video (n.g.)	40 min: 5 reps × 6 min set, 42 type compositions each, 2 min rest (more details n.g.)
Woo, 2007	Healthy > 8 y post Sedentary	12	No	Tai Chi (Yang-Style)	? Yes	3 × ?, S-JE (81%)	24 forms of Yang-Style Tai Chi
Wu, 2006	Healthy 4 ± 2 y post Sedentary	12	No	Walking	No Yes	3 × 60, S-JE (n.g.)^*^	45 min of walking with 5–6 km/h
Yamazaki, 2004	≥ Osteopenia 17 ± 8 y post Sedentary	12	No	Walking	No Yes	≥ 4 × 60, n.g. (presumably HE) (100%)	8000 steps/session at 50% VO_2_max
*DRT type exercise (n = 18)*							
Bemben, 2000	Healthy 3 ± 1 y post No-RT	6	Yes	DRT (all main muscle groups) with machines	Yes Yes	3 × 60, S-JE (87%)	DRT:45 min, 8 exercises, 3 sets, 8 reps, 80% 1RM
		6	Yes	DRT (all main muscle groups) with machines	Yes Yes	3 × 60, S-JE (93%)	DRT: 45 min, 8 exercises, 3 sets, 16 reps, 40% 1RM
Chilibeck, 2002	Healthy 9 ± 2 y post No vigorous Ex	12	Yes	DRT (all main muscle groups) on machines	Yes Yes	3 × ?, S-JE (78%)	12 exercises, 2 sets, 8–10 reps, ≈ 70% 1RM
De Oliveira, 2018	Healthy 8 ± 7 y post Sedentary	6	Yes	Pilates (all main muscle groups) on machines	Yes Yes	3 × 60, S-JE (93%)	21 exercises (strengthening & flexibility), 1 set, 10 reps, 1 min rest between exercises, 5–6 at Borg CR10
Duff, 2016	Healthy > 8 y post No-RT	9	Yes	DRT (all main muscle groups) on machines & with free weights	Yes Yes	3 × ?, S-JE (84%)	12 exercises, 2 sets, 8–12 reps to muscular fatigue, ? 1RM (more details n.g.)
Hartard, 1996	Osteopenia > 2 y post < 1 h/w, No-BSE	6	Yes	DRT (all main muscle groups) on machines	Yes Yes	2 × ?, S-JE (> 83%)	14 exercises, 1–2 sets, 8–12 reps, 70% 1RM, TUT: concentric: 3-4 s – eccentric 3-4 s. ≥ 2 min rest between sets
Kerr, 1996	Healthy ≈ 7 ± 4 y post No-RT, No-Ex > 3 h/w	12	Yes	Unilateral DRT (all main muscle groups, randomized allocation of the left side or right side to exercise or control group) on machines or free weights	Yes Yes	3 × 45–60, S-JE (89%)	13 exercises, 3 sets at 20 RM, 3–5 rep (≈ 60–65% 1RM), 2–3 min rest between sets
		12	Yes	Unilateral DRT (see above)	Yes Yes	3 × 20–30, S-JE (87%)	13 exercises, 3 sets at 8 RM, 3–5 rep (≈75–80% 1RM), 2–3 min rest between sets
Maddalozzo, 2007	Healthy 1–3 y post n.g	12	Yes	DRT (back squat, deadlifts) with free weights	Yes Yes	2 × 50, S-JE (85%)	15–20 min warm up (exercise focusing on posture, muscle engagement, abdominal strength, flexibility) 2 sets, 10–12 reps, 50% 1 RM. Main part: 20–25 min, 2 exercises, 3 sets, 8–12 reps, 60 s rest between sets at 60–75% 1RM, TUT: 1-2 s concentric, 2-3 s eccentric
Nelson, 1994	Healthy (6 women with 1 spine fracture) 12 ± 5 y post Sedentary	12	Yes	DRT (most main muscle groups) on machines	Yes Yes	2 × 55, S-JE (88%)	45 min, 5 exercises, 3 sets, 8 reps, 50- 80% 1RM, TUT-6–9 s/rep, 3 s rest between reps, 90–120 s rest between sets
Nicholsen, 2015	Healthy> 5 y post No-RT	6	Yes	DRT (all main muscle groups): „Body Pump Release 83 “ (i.e., barbell exercises)	Yes Yes	2 × 50, S-JE (89%)	10 × up to 6 min blocks of exercises for all main muscle groups (21 exercises in total); up to 108 reps (squats), ≤ 30% 1RM
Orsatti, 2013	Healthy 9 ± 6 y post Sedentary	9	Yes	DRT (all main muscle groups) with free weights and on machines	Yes Yes	3 × 50–60, S-JE (n.g.)	8 exercises 3 sets, 8–15 reps at 40–80% 1RM, 3 sets -20–30 reps for trunk flexion & calf raises, 1–2 min rest between sets
Pruitt [28], 1992	Healthy 3 ± 1 y post No-BSE	9	Yes	DRT (all main muscle groups) with free weights and on machines	Yes Yes	3 × 60, S-JE (83%)	40 min, 11 exercises, 1 set, at 10 -12 RM for upper body & 10–15 RM for lower body (more details n.g.)
Pruitt, 1995	Healthy > 8 y post No-RT	12	Yes	DRT (all main muscle groups) on machines	Yes Yes	3 × 55–65, S-JE (81%)	50–55 min, 10 exercises, 1 warm up set, 14 reps, at 40% 1 RM, 2 sets, 7 reps, 80% 1RM
	Healthy > 8 y post No-RT	12	Yes	DRT (all main muscle groups) on machines	Yes Yes	3 × 55–65, S-JE (77%)	50–55 min, 10 exercises, 3 sets, 14 reps, at 40% 1RM
Sinaki, 1989	Healthy > 0.5 y post n.g	24	Yes	DRT (back strengthening exercise in a prone position using a back pack; ≈ hyperextensions) with free weights	Yes No	5 × ?, HE (n.g.)	One back strengthening exercise, 1 set, 10 reps, with a weight equivalent to 30% of the maximum isometric back muscle strength in pounds (maximum 23 kg)
Wang, 2015	Healthy > 0.5 y post No Tai Chi	12	No	Tai Chi-RT (includes 4 resistance based Chen style actions)	? Yes	2 × 60, S-JE 2 × 60, Group E with video (n.g.)	40 min: 6 reps × 5 min exercise, 2 min rest (more details n.g.)
Woo, 2007	Healthy > 8 y post Sedentary	12	No	DRT (arm-lifting, hip abduction, heel raise, hip-flexion,-extension, squat) with elastic bands	Yes Yes	3 × ?, S-JE (76%)	6 exercises, 30 reps (no more information given)
*Combined WB und DRT (n = 36)*							
Adami, 1999	Healthy 16 ± 7 y post Sedentary	6	No	Walking, DRT; volleyball in a sitting/standing position	No Yes	2 × 95–110, S-JE (83%) 7 × 30 HE (n.g.)	S-JE: 15–30 min warm up (walking), 70 min press-up, volleyball, 10 min DRT for the forearm with a 500 g weight. Number of reps (10–25)/min increased progressively. HE: Repeat all exercise
Basat, 2013	Osteopenia 6 ± 4 y post No-BSE	6	No	Walking, DRT (focus on lower body with few trunk exercises)	Yes Yes	3 × 60, S-JE (> 60%)	15 min warm up (walking), 30–40 min RT: ≥ 9 exercises, 1 set, 10 reps (more details n.g.)
Bello, 2014	Diabetes 61 ± 6 y Low intensity	8	No	Walking; DRT (all main muscle groups); aquatic exercise (RT main muscle groups)	Yes Yes	3 × 40-?, S-JE (85%)	40 min walking 1 × w, WB circuit training 1 × w with easy loads: 6 exercises, 3 sets, 15–20 reps. Aquatic RT exercise 1 × w: 4 exercise, 3 sets, 15–20 reps; all at RPE 12–15 of Borg CR 20. 1 × week each type of exercise
Bemben, 2010	Healthy > 5 y post No-RT	8	No	Walking, DRT (all main muscle groups) with machines	Yes Yes	3 ×≈ 60, S-JE (90%)	5 min warm up (walking), 8 exercises, 3 sets, 10 reps, 80% 1RM + dumbbell wrist curls & seated abdominal flexion L/M intensity
Bergström, 2008	Osteopenia 59 ± 4 y No-BSE	12	Yes	Walking, DRT (all main muscle groups);	Yes Yes	1–2 × 60, S-JE 3 × 30, HE HT & S-JE (95%)	S-JE: 25 min DRT, 25 min WB AET (more details n.g.) HE: fast walking (more details n.g.)
Bocalini, 2009	Healthy > 8 y post Sedentary	6	Yes	Running, DRT (all main muscle groups)	Yes Yes	3 × 60, S-JE (> 90%)	10 min warm up (low impact running), 12 exercises, 3 sets, 10 reps, 85% 1RM with focus on eccentric exercises, 1 min rest (alternate upper & lower body exercises) between ex
Bolton, 2012	Osteopenia 13 ± 7 y post No-BSE	12	Yes	Jumping, DRT (muscle groups n.g.: “loading the proximal femur”)	No Yes	3 × 60, S-JE 1/w (88%) Daily HT	S-JE: 40 min (?) exercises, 2 sets, 8 reps, 80% 1RM with slow velocity, 1 set with reduced load & high velocity (12 rep). HT: Daily 3 sets, 10 reps of jumps (more details n.g.)
Caplan, 1993	Healthy 18 ± 8 y post n.g	24	No	Aerobic dance, ball games; DRT: floor exercises (more details n.g.)	? Yes	2 × 60, S-JE (n.g.) ≥ 1 × 20–30, HT (n.g.)	20–25 min AET, 10 min ball games (more details n.g.) 20–30 min DRT (more details n.g.)
Chilibeck, 2013	Healthy > 1 y post No-BSE	24	Yes	Walking; DRT (all main muscle groups) on machines	Yes Yes	2 × n.g., S-JE 4 × 20–30, HT & S-JE (77%)	S-JE: 15 exercises, 2 sets, 8 reps, 80% 1RM HT & S-JE: walking at 70% HRmax
Choquette, 2011	Healthy 8 ± 8 y post Sedentary	6	Yes	Running & cycling; DRT (all main muscle groups) on machines & with free weights	Yes Yes	3 × 60, S-JE (≥ 85%)	AET: 30 min at 40–85% HRmax; after 3 months H-intensity intervals of 4 × 4 min ≥ 90% HRmax, 3 min rest at 50–65% HRmax. RT: 30 min, ? exercise, 1 set, 12–15 rep increased to 4 sets 4–6 reps, at 60%-85%1RM
Chuin, 2009	Healthy > 8 y post n.g	6	Yes	Running & cycling, DRT (most main muscle groups) on machines	Yes Yes	3 × 60, S-JE (> 90%)	15 min warm up (treadmill/cycle ergometer), DRT: 45 min, 8 exercises, 3 sets, 8 reps at 80% 1RM, rest between sets 90–120 s, 1 RM-test each 4 weeks
De Matos, 2009	≥ Osteopenia 10 y post n.g	12	Yes	Running, stepping, cycling, DRT (all main muscle groups) on machines or free weights;	Yes Yes	3 × 45–65, n.g. (presumably S-JE) (n.g.)	WB / non-WB AET (treadmill, stepper, bike): 5–20 min (RPE 4–6 on Borg CR 10). DRT: 30–40 min, 9 exercises,? sets, 10–15 reps, ? 1RM, TUT: 3 s conc-3 s eccentric; 1 min rest between sets & exercise
Deng, 2009	Healthy 4 ± 3 y post No-BSE	12	Yes	Brisk walking, stepping, jumping; DRT (all main muscle groups) on machines with free weights	Yes Yes	2 × 60, S-JE 3–5 × 60, HE (82%)	S-EJ: 45 min DRT, 9 exercises, 2–5 sets, 12–40 reps, at 50–60% 1RM, self-selected rest (more details n.g.) HE: 30 min walking, at 50–80% HRmax, 15 min step routine, 50–300 jumps from a 4 inch bench
Englund, 2005	Healthy > 8 y post n.g	12	Yes	Walking/jogging; DRT (all main muscle groups)	Yes Yes	2 × 50, S-JE (67%)	WB AET: 10 min warm up, 15 min walking/jogging. DRT: 12 min, 2 sets, 8–12 reps., ? 1RM (more details n.g.)
Going, 2003	Healthy 3–11 y post No-RT, < 120 min Ex	12	Yes	Walking, Jogging, skipping, hopping, stepping with weighted vests; DRT (all main muscle groups) on machines with free weights	Yes Yes	3 × ≈ 60, S-JE (72%)	10 min warm up (walking), 20–25 min WB AET at 60% HRmax, 120–300 stair/steps with 5- 13 kg weighted vest DRT: 7 exercises, 2 sets, 6–8 reps 70–80% 1 RM
Grove, 1992	Healthy 4 ± 3 y post Sedentary	12	No	Jumping variations, heel drops (GRF ≥ 2 × body mass); DRT	? Yes	3 × 60, S-JE (83%)	20 min of high impact exercises. 15 min cool down (RT with abdominal & leg adduction/abduction exercises)
		12	No	Walking, charleston, heel jacks (GRF < 1.5 × body mass), DRT	No Yes	3 × 60, S-JE (80%)	20 min of low impact exercises. 15 min cool down (RT with abdominal & leg adduction/abduction exercises)
Iwamoto, 2001	Osteoporosis 16 ± 6 y post Sedentary	24	Yes	Walking; DRT (“Gymnastics”: lower limbs & trunk exercises)	Yes Yes	Daily (walking), ?, HE 2 × daily RT?, HE (n.g.)	Additionally (to basic activity walking) ≈ 3000 steps/d, RT: ≥ 4 exercises, 2 sets, 15 reps, ?% 1RM
Jessup, 2003	Healthy > 8 y post Sedentary	8	Yes	Walking, stairclimbing; DRT (most main muscle groups) on machines	Yes Yes	3 × 60–90, S-JE (n.g.)	DRT: 20–35 min, 8 exercises, ? sets, 8–10 reps, 50%- 75% 1RM. WB AET: 30–45 min with weighted vest (increased up to 10% body mass)
Karakiriou, 2011	Osteopenia 5 ± 2 y post Sedentary	6	No	Walking, jumping, step aerobic exercise; DRT (all main muscle groups)	Yes Yes	2 × ? RT, S-JE 1 × 45 min AET (80%)	15 min warm up (walking, cycling, jumping). 2 × RT/w.:11 exercises, 2–3 sets, 10–12 reps at 70% 1RM, 30 s rest between exercises, 3 min between sets. 1 × Step aerobic/w.: 20 min, 9 exercise, 2 circuits of 40 s; rest: 20 s between exercises, 2 min between circuits, 70–85% HRmax
Kemmler, 1999	Healthy Early-post-menopausal No-BSE	9	Yes	Running, gaming, jumping; DRT (all main muscle groups)	Yes Yes	2 × 90, S-JE (82%) 2 × 35, HT (59%)	AET: 25 min at 70–80% HRmax. RT: 65 min, 12–15 exercises, 2–4 sets of 8 s maximum isometric contractions; 6 trunk, upper back, lower extremity exercises, 20–25 reps at 60–65% 1 RM. HT: resistance exercises
	Healthy Late-post-menopausal No-BSE	9	Yes	Running, gaming, jumping; DRT (all main muscle groups)	Yes Yes	2 × 90, S-JE (82%) 2 × 35, HT (59%)	AET: 25 min at 70–80% HRmax. RT: 65 min, 12–15 exercises, 2–4 sets of 8 s maximum isometric contractions; 6 trunk, upper back, lower extremity exercises, 20–25 reps at 60–65% 1 RM. HT: resistance exercises
Kemmler, 2004	Osteopenia 1–8 y post No-BSE	26	Yes	Running, HI-aerobic dance jumping; DRT (all main muscle groups) on machines, with free weight, body mass	Yes Yes	2 × 60–70, S-JE (79%) 2 × 25, HT (61%)	AET: 20 min at 65–85% HRmax. Jumping started after 5–6 months with 4 × 15 multilateral jumps. DRT: 30–40 min, 1/w. The first 6 month: 13 ex, 2 sets, 20–12 rep, TUT: 2 s concentric, 2 s eccentric at 50–65% RM, 90 s rest between sets & exercises. Then, 12 w blocks of H-intensity at 70–90% 1RM interleaved by 4 w at 55–79% 1RM. Isometric RT: 30–40 min, 1/w, 12–15 exercises (trunk& femur), 2–4 sets, 15–20 rep, 15–20 s rest. HT: rope skipping (3 set, 20 rep), RT
Kemmler, 2010	Healthy > 8 y post Sedentary	18	Yes	HI-aerobic dance; DRT (all main muscle groups)	Yes Yes	2 × 60, S-JE (76%) 2 × 20, HE (42%)	AET: 20 min at 70–85% HRmax. RT: 10–15 exercises, 1–3 sets of 6–10 s maximum isometric contractions, 20–30 s rest, 3 upper body exercises, 2–3 sets 10–15 reps, TUT: 2 s concentric, 2 s eccentric at 65–70% 1RM; 3 lower extremity exercises, 2 sets 8 reps, 1 min rest at 80% 1RM. HT: RT 1–2 sets, 6–8 exercise, 10–15 rep. 2–3 belt exercises, 2 sets, 10–15 rep
Kemmler, 2013	Healthy 2 ± 1 y post No-BSE	12	Yes	HI-aerobic dance, walking/ running, jumping; isometric & DRT (all main muscle groups) exercise on machines, with free weight, body mass	Yes Yes	3 × 45–60, S-JE (67%)	Block I: 1 × 45 min/w H-Impact aerobic 75–85% HRmax, 2 × 20 min/w aerobic 75–85% HRmax, 4 × 15–20 jumps, 90 s rest. RT: 15 min, 8–12 floor exercises (trunk, hip, legs), 1–2 sets, 10–20 rep, 30 s rest. RT: 20 min, 8 exercises, 2 sets, 8-9rep, 45 s rest up, TUT: 2 s concentric, 2 s eccentric. to 80% 1RM
Kerr, 2001	Healthy ≈10 ± 6 y post < 2 h/w	24	Yes	Walking, DRT (all main muscle groups)	Yes Yes	3 × 60, S-JE (74%)	≈ 30 min brisk walking & stretching, RT: 30 min, 9 exercises, 3 sets at 8 RM (≈ 75–80% 1RM)
		24	No	Walking, DRT (all main muscle groups); Stationary cycling	Yes Yes	3 × 60, S-JE (77%)	≈ 30 min brisk walking & stretching. RT: 30 min, 9 exercises, 3 set, 8 rep, 40 s/exercise with “minimal load”; 10 s rest between the exercises (more details n.g.). Stationary cycling 40 s, HR < 150 beats/min
Kwon, 2008	Healthy > 8 y post No-Ex > 2/w	6	Yes RT?	Aerobic dance; DRT (6 upper & lower body exercises) with free weights	Yes Yes	3 × 80, n.g. (presumably S-JE) (n.g.)	30 min AET at 40–75% HRmax, 30 min DRT of 6 exercises, ? sets, 3–10 reps to voluntary fatigue (i.e., 75% 1RM)
Lord, 1996	Healthy > 8 y post No equal intensity with the intervention	12	No	Brisk walking, multilateral stepping, lunges, heel rises; DRT (all main muscle groups) using owns body mass	Yes Yes	2 × 60, S-JE (73%)	5 min warm up (paced walking), conditioning period 35–40 min: AET & guided functional gymnastics for all main muscle groups (sets?, reps?, intensity?)
Marques and Mota, 2011	Healthy > 8 y post Sedentary	8	Yes	Marching, bench stepping, heel drops; DRT (most main muscle groups) with weighted vests, elastic bands, free weights	Yes Yes	2 × 60, S-JE (72%)	15 min WB AET with Peak-GRF up to 2.7 × body mass & high strain frequency (120–125 beats/min), 10 min for ≥ 7 muscle endurance exercises, 1–3 sets, 8–15 reps, ?1RM (more details n.g.), 10 min balance & dynamic exercise (walking, playing with ball, rope, sticks, etc.), 10 min agility training (coordination, balance, ball games, dance)
Marques and Wanderley, 2011	Healthy > 8 y post Sedentary	8	Yes	DRT (all main muscle groups) on machines, walking during cool down	Yes Yes	3 × 60, S-JE (78%)	8–10 min warm up (cycling/rowing ergometer) at low intensity. 30–40 min DRT, 8 exercises, 2 sets, 15–6 reps, 50–80% 1RM with variable TUT (3-6 s/rep.), 120 s rest between sets, 5–10 min cool down (walking & stretching)
Milliken, 2003	Healthy 6 ± 3 y post < 2 h/w	12	Yes	Walking, skipping, multilateral stepping, jumping with weighted vests; DRT (all main muscle groups) with free weights, on machines; functional gymnastics	Yes Yes	3 × 75, S-JE (n.g.)	20 min WB AET at 50–70% HRmax. 35 min DRT: 8 exercises, 2 sets, 6–8 reps, 70–80% 1 RM. Functional gymnastics for shoulder & abdominals using elastic bands and physio-balls
Nichols, 1995	Healthy > 8 y post ≥ 3 × 30 min/w	12	Yes	Walking, DRT (all main muscle groups) on machines	Yes Yes	3 × ≈ 45–60, S-JE (82%)	5 min warm up (walking), 8 exercises, 1–3 sets, 10–12 reps, 50–80% 1RM; 30-60 s rest between exercises, 60 s rest between sets
Park, 2008	Healthy > 8 y post ≤ 7 h/w M-Ex	12	No	WB AET; RT (more details n.g.)	? Yes	3 × 60, n.g (n.g.)	10 min RT, 23 min of WB exercise at 65–70% HRmax (more details n.g.)
Tolomio, 2009	≥ Osteopenia 2–22 y post n.g	11	No	Walking, DRT (joint mobility, elastic bands, balls); aquatic exercise (more details n.g.)	? Yes	3 × 60, S-JE & 1 × HE (n.g.)	The first 11 w only in gym, then 2 times in gym & once in water. 15 min warm up (brisk walking, stretching), 2 × 30 min/week RT, 1 × 30 min/week water gymnastics (more details n.g.). 2 periods (6 &10 w) training at home (more details n.g.)
Verschueren, 2004	Healthy 15 ± 6 y post n.g	6	Yes	Running, Stepping, DRT (leg press, leg extension)	? Yes	3 × 60, n.g. (presumably S-JE) (n.g.)	20 min warm up (running, stepping, or cycling) at 60–80% HRmax, DRT:2 exercise, 1–3 set, 20–8 rep

*AET* aerobic exercise training, *BSE* bone-specific exercise, *DRT* dynamic resistance training, *GRF* ground reaction forces, *HE* home exercise, *JE* joint exercise program, *PS* partially supervised, *PR-INT* progression of intensity parameters, *PrInt* progression of intensity, *RPE* rate of perceived exertion, *S* supervised, *WB* weight bearing, *TUT* time under tension, *L* low, *M* moderate, *H* high

Status: With few exceptions, we focus on osteoporosis/osteopenia and fractures. Period of menopausal status: In the case of no information, the mean age was reported. Physical activity: Predominately we used the characterization of the authors. In some cases (e.g., Martin 1993) we summarize the information given to “no bone-specific exercise” (no-BSE). Progression: We only consider the progression of exercise intensity. Type of exercise: We subsume the information given in weight bearing (WB) vs. Non-WB aerobic exercise training (AET); resistance (RT) or dynamic resistance exercise (DRT), jumping, aquatic exercise or Tai Chi; Site specificity: First line: Estimated site specific of the exercise type on LS-BMD; Second line: Estimated site specific of the exercise type on FN-BMD. Exercise volume/week; setting, attendance: Number of sessions per week × minutes per session (e.g., 3 × 60); setting of the exercise application, i.e., either supervised group exercise (S-JE) or home exercise or exercise individually performed without supervision (HE). In parenthesis: Attendance as defined as rate of sessions performed (%). Composition of strain/exercise parameters per session: AET: specific exercise (i.e., walking, jogging, aerobic dance), exercise duration, exercise intensity; DRT: exercises/number of exercises; number of sets, number of repetitions; exercise intensity; jumping: type of jumps, number of jumps, intensity of jumps; Tai Chi: style, number of forms. We did not include warm up in the table, if the authors did not report the duration and type of exercise as warm up; cycle ergometer ≤ 5 min as warm up, stretching and balance as cool down have not been included in the table

^a^Presumably low, according to the additional number steps/day compared with the sedentary control group

### Intervention Characteristics

#### Cholecalciferol, Calcium Supplementation

Vitamin-D and/or calcium supplementation for the exercise and control groups were provided in 21 studies [18, 19, 21, 29, 33–35, 40, 41, 43, 44, 49, 51, 65, 71, 73, 76, 77, 79, 80, 84].

#### Exercise Intervention

Table 2 gives exercise characteristics of the included studies categorized according the type of exercise.

#### Dynamic Resistance Exercise (DRT)

Table 2 (midsection) specifies the exercise protocols of included 18 included DRT study groups.

Apart from one exception [30], all studies focused on a supervised group exercise protocol [18–29, 31, 32, 60]. Studies ranged from six [18, 20, 22, 26] to 24 months [30]. Except for three studies/study groups [24, 30, 32], the studies focused on all or most main muscle groups. Besides two studies that did not provide sufficient information for the LS-site [23, 32], all the other studies provided exercises for their specified BMD-ROI (i.e., LS and proximal femur). Most studies prescribed a training frequency of three sessions per week (Table 2); with a session length that varied from about 1–2 min (i.e., 10 × back extension [30]) to ≈ 120 min (39 sets × 20 reps, 2–3 min of rest [23]). Most studies applied a multiple set approach ( [18, 19, 21–25, 27, 29, 43, 60]. Relative exercise intensity ranged between 80% 1RM [18, 23, 25, 27, 29, 43, 60] and ≤ 30% 1RM [26, 30]. Five studies prescribed either work to repetition maximum [27, 28, 80] or work to muscular fatigue [21, 43], another study [20] referred to 5–6 (i.e., strong-strong +) on the Borg CR10 scale. Reviewing the repetition number and relative exercise intensity (% 1RM), some studies [30] or study arms [18, 29] might have exercised with (too) low effort.^1^ None of the studies reported an explosive movement in the concentric or eccentric phase. Progression or at least regular adjustments of exercise intensity were realized by all but one DRT study. Periodization models [90] were not applied by any of the studies.

#### Weight Bearing Exercise

Table 2 (upper part) lists the exercise protocol of the weight bearing type exercise studies. By nature, the specific exercise was much more heterogeneous compared with DRT. Studies specified (brisk) walking including walking with additional load (*n* = 11), walking/running (*n* = 3), Tai Chi (*n* = 4), jumping or rope skipping (*n* = 3), heel drops (*n* = 1), stepping (*n* = 1), standing on one leg (*n* = 1) and combined weight bearing types (e.g., heel drops, jumping skipping; stairclimbing, *n* = 6) (Table 2). Duration of the studies varied between 6 (e.g., [33].) and 30 months [45]. Twelve of 30 study groups applied a supervised group exercise program, 12 study groups specified non-supervised individual exercise [36, 37, 39, 41, 42, 44, 47, 53, 55, 89] or additional [34, 35, 45, 51, 89] to the supervised group exercise, for 6 study groups this information was not listed (Table 2). Site specificity at the LS might be realized by direct muscular insertion of exercises applied in the Tai Chi studies [31, 32, 38, 47] and studies that applied higher ground reaction forces (i.e., jumping, drop-jumps and potentially jogging in older cohorts) [33–35, 40, 41, 43–46, 52, 55, 56, 83]. Net training frequency (considering attendance rate) varied between ≥ 10 sessions^2^ and about 2 sessions/week [55]; corresponding net exercise volume/week vary between ≥ 240 [89] and about 10–15 min/week [55]. Progression of exercise intensity was consciously considered by about half of the WB type exercise studies [40, 41, 43–46, 49, 52, 56, 83] (Table 2). Periodized exercise models were not applied.

#### Combined WB and DRT Studies

Most combined WB DRT studies applied a combination of walking, running, stepping, movement games, dancing either as single session, session component or during warm up and a DRT on machines or with free weights (Table 2, lower part). At least nine study arms [63, 69, 72, 76, 77, 79, 82, 84, 91] specified exercises with higher GRF (e.g., jumping variations, heel drops) during the WB&DRT sessions. With few exceptions [73], the studies scheduled either a consistently supervised exercise protocol or a mix of supervised (DRT) sessions and non-supervised walking/home training sessions (Table 2). Duration of the studies varied between 6 (e.g., [33, 58].) and 26 months [77]. Training frequency varied from ≈ 8 [58] to < 2 sessions/week [70, 82, 83]; net training volume ranged from about 6 h/week [58] to 67 min/week [70]. Due to the overall DRT (i.e., all or most main muscle groups) that was applied by all, but 3 studies [58, 72, 88], most studies mechanically addressed the LS-ROI by muscular tension. Apart from one study [66], all studies that adequately described their exercise protocols applied multiple set approaches. Peak relative exercise intensity varied between 90% 1RM [77] and 60–65% 1RM [76]. Progression of exercise intensity was applied by the vast majority of the studies [48, 62, 63, 65–71, 73, 74, 76–81, 83–85, 88].

### Methodologic Quality

The Pedro scores of the included studies are listed in Table 3. Methodologic quality of the trials ranges from 3 to 9 score points (Table 3), with a mean and SD of 5.44 ± 1.32 score points. Methodologic quality of the DRT studies was on average (6.24 ± 1.30 points) significantly higher (*P* = 0.024) compared with the other groups.

**Table 3:** Assessment of risk of bias for included studies listed in alphabetic order

First author, year	Eligibility criteria	Random allocation	Allocation concealment	Inter group homogeneity	Blinding subjects	Blinding personnel	Blinding assessors	Participation ≥ 85% allocation	Intention to treat analysis^a^	Between group comparison	Measure of variability	Total score
Adami, 1999	Y	0	0	1	0	0	0	1	0	1	1	4
Basat, 2013	Y	1	1	1	0	0	0	0	0	1	1	5
Bassey, 1995	Y	1	0	1	0	0	0	0	0	1	1	4
Bassey, 1998	Y	1	0	1	0	0	0	0	0	1	1	4
Bello, 2014	Y	1	0	1	0	0	0	0	1	1	1	5
Bemben, 2000	Y	1	0	1	0	0	0	0	0	1	1	4
Bemben, 2010	Y	0	0	1	0	0	0	1	1	1	1	5
Bergström, 2008	Y	1	1	1	0	0	0	0	1	1	1	6
Bocalini, 2009	Y	1	0	1	0	0	1	0	0	1	1	5
Bolton, 2012	Y	1	1	0	0	0	1	1	1	1	1	7
Brooke-Wavell, 1997	Y	1	0	1	0	0	0	1	0	1	1	5
Brooke-Wavell, 2001	Y	0	0	1	0	0	0	1	1	1	1	5
Caplan, 1993	Y	0	0	1	0	0	0	1	1	1	1	5
Chan, 2004	Y	1	0	1	0	0	0	0	1	1	1	5
Chilibeck, 2002	Y	1	1	1	1	1	0	0	1	1	1	8
Chilibeck, 2013	Y	1	1	1	0	0	1	1	1	1	1	8
Choquette, 2011	Y	1	0	1	0	0	0	0	1	1	1	5
Chuin, 2009	Y	1	0	1	0	0	0	0	1	1	1	5
de Matos, 2009	Y	0	0	1	0	0	0	0	0	1	1	3
Deng, 2009	Y	0	0	1	0	0	0	1	0	1	1	4
de Oliveira, 2019	Y	1	1	1	0	0	1	1	1	1	1	8
Duff, 2016	Y	1	1	1	1	0	1	0	1	1	1	8
Ebrahim, 1997	Y	1	1	1	0	0	1	0	1	1	1	7
Englund, 200	Y	1	0	1	0	0	0	0	0	1	1	4
Evans, 2007	Y	1	1	1	0	0	0	0	1	1	1	6
Going, 2003	Y	1	0	1	0	0	0	0	1	1	1	5
Grove, 1992	Y	1	0	1	0	0	0	1	1	1	1	6
Hans, 2002	Y	1	0	1	0	0	0	0	1	1	1	5
Hartard, 1996	Y	0	0	1	0	0	0	1	1	1	1	5
Hatori, 1993	Y	1	0	1	0	0	1	1	0	1	1	6
Iwamoto, 2001	Y	1	0	1	0	0	0	0	1	1	1	5
Jessup, 2003	Y	1	1	0	0	0	1	1	1	1	1	7
Karakiriou, 2011	Y	1	0	0	0	0	0	0	0	1	1	3
Kemmler, 1999	Y	0	0	1	0	0	0	1	1	1	1	5
Kemmler, 2004	Y	0	0	1	0	0	0	0	1	1	1	4
Kemmler, 2010	Y	1	1	1	1	0	1	1	1	1	1	9
Kemmler, 2013	Y	1	0	1	1	0	1	0	1	1	1	7
Kerr, 1996	Y	1	0	1	0	0	0	0	1	1	1	5
Kerr, 2001	Y	1	0	1	0	0	0	0	1	1	1	5
Kohrt, 1995	Y	0	0	1	0	0	0	0	1	1	1	4
Kohrt, 1997	Y	0	0	1	0	0	0	0	1	1	1	4
Korpelainen, 2006	Y	1	1	1	0	0	1	0	1	1	1	7
Kwon, 2008	Y	0	0	1	0	0	0	0	0	1	1	3
Lau, 1992	Y	1	1	1	0	0	0	0	1	0	1	5
Liu, 2015	Y	1	0	1	0	0	0	1	1	1	1	6
Lord, 1996	Y	1	0	1	0	0	0	0	1	1	1	5
Maddalozzo, 2007	Y	1	0	1	0	0	0	1	1	1	1	6
Marques, 2011	Y	1	1	1	0	0	0	0	1	1	1	6
Marques, 2011	Y	1	1	1	0	0	0	0	1	1	1	6
Martin, 1993	Y	1	0	1	0	0	0	0	1	1	1	5
Milliken, 2003	Y	1	0	1	0	0	0	1	1	1	1	6
Nelson, 1991	Y	0	0	1	0	0	0	1	1	1	1	5
Nelson, 1994	Y	1	0	1	0	0	0	1	1	1	1	6
Nichols, 1995	Y	1	0	1	0	0	0	0	1	1	1	5
Nicholson, 2015	Y	1	1	1	0	0	0	1	1	1	1	7
Orsatti, 2013	Y	1	1	1	0	0	0	1	1	1	1	7
Park, 2008	Y	1	1	1	0	0	0	1	1	1	1	7
Prince, 1995	Y	1	1	1	0	0	0	0	1	1	1	6
Pruitt, 1992	Y	0	0	1	0	0	0	1	1	1	1	5
Pruitt, 1995	Y	1	0	1	0	0	0	0	1	1	1	5
Ryan, 1998	Y	0	0	1	0	0	0	0	1	1	1	4
Sakai, 2010	Y	1	1	1	0	0	0	1	0	1	1	6
Silverman, 2009	Y	0	0	1	0	0	1	0	0	1	1	4
Sinaki, 1989	Y	1	0	1	0	0	0	1	1	1	1	6
Sugiyama, 2002	Y	0	0	1	0	0	0	0	0	1	1	3
Tartibian, 2011	Y	1	0	1	0	0	0	1	1	1	1	6
Tolomio, 2009	Y	1	0	1	0	0	0	1	0	1	1	5
Verschueren, 2003	Y	1	1	1	0	0	1	0	0	1	1	6
Wang, 2015	Y	1	0	1	0	0	0	1	1	1	1	6
Woo, 2007	Y	1	1	1	0	0	1	1	1	1	1	8
Wu, 2006	Y	1	0	1	1	0	0	0	1	1	1	6
Yamazaki, 2004	Y	0	0	1	0	0	0	0	1	1	1	4

^a^The point is awarded either for intention to treat analysis or when “all subjects for whom outcome measures were available received the treatment or control condition as allocated”

### Outcomes

Apart from two studies [28, 30] that applied DPA, all the others used DXA. Furthermore, all the other studies except two ( [23]: hip only; [30]: LS only) determined both, BMD at LS and proximal femur regions of interest.

### Effect of Different Types of Exercise on LS-BMD

**Fig. 2:**
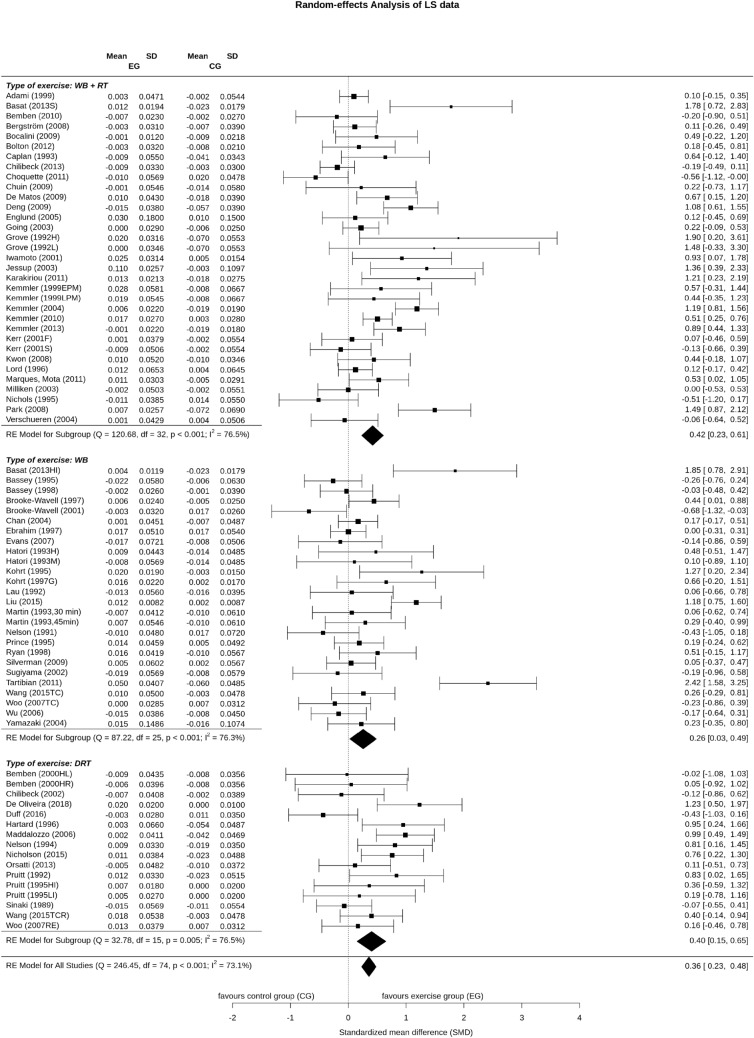
Forest plot of meta-analysis results at the LS. The data are shown as pooled standard mean difference (SMD) with 95% CI for changes in exercise and control groups

Sixteen DRT exercise groups, 26 WB exercise groups and 33 combined WB&DRT exercise groups evaluated the effect of exercise on LS-BMD. In summary, the pooled estimate of random effect analysis for DRT was SMD: 0.40, 95% CI 0.15–0.65 (*P* = 0.009), for WB exercise SMD: 0.26, 95% CI: 0.03–0.49 (*P* = 0.037) and SMD: 0.42, 95% CI 0.23–0.61 (*P* = 0.001) for the combined WB&DRT exercise. No significant differences between the types of exercise were observed (*P* = 0.508). All types of exercise revealed a similarly high level of heterogeneity between their trials (*I*^*2*^ = 76.3–76.5%)) (Fig. 2).

### Effect of Different Types of Exercise on FN-BMD

**Fig. 3:**
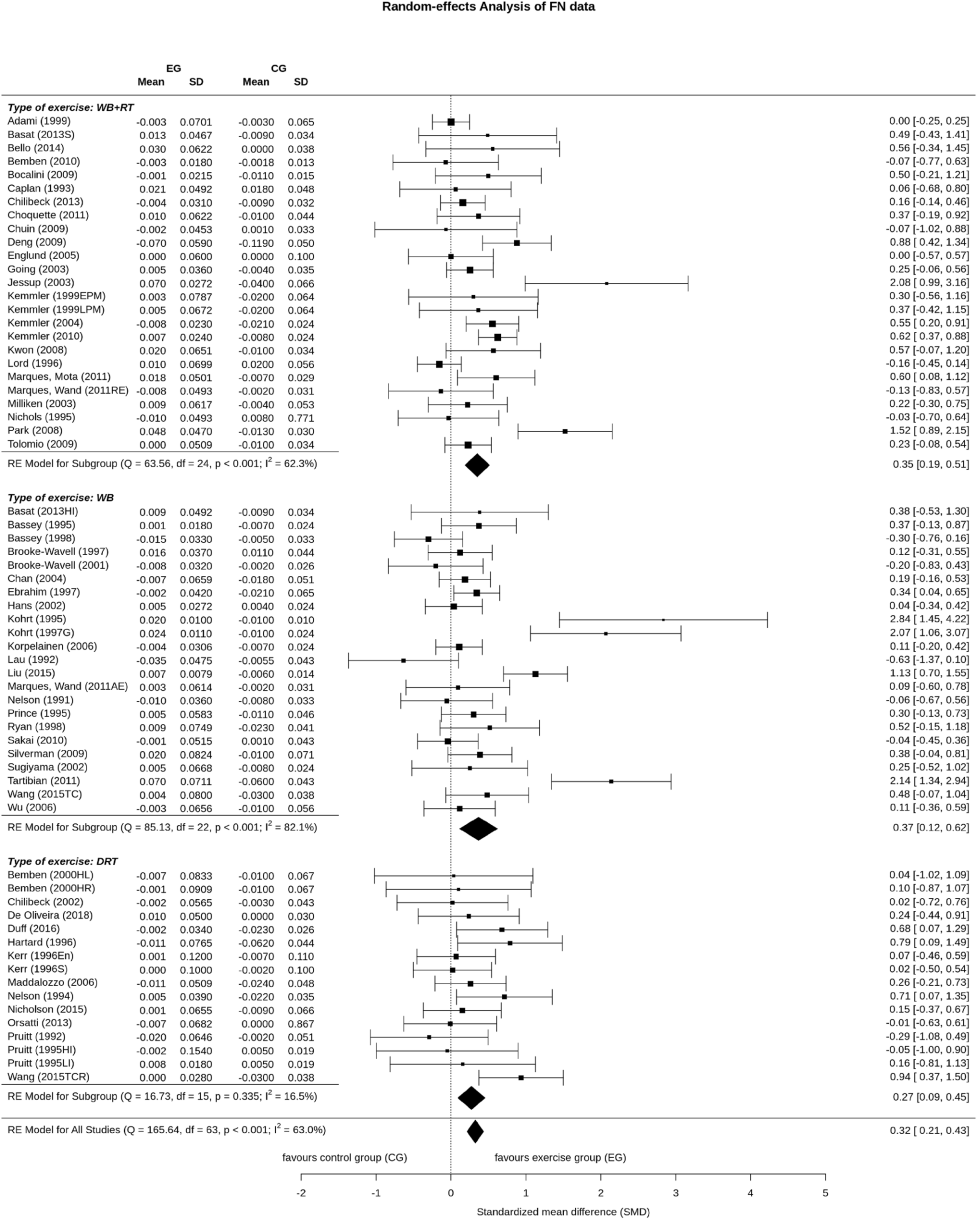
Forest plot of meta-analysis results at the femoral neck. The data are shown as pooled standard mean difference (SMD) with 95% CI for changes in exercise and control groups

Fifteen DRT exercise groups, 23 WB exercise groups and 25 combined WB&DRT exercise groups evaluated the effect of exercise on femoral neck-BMD. In summary, the pooled estimate of random effect analysis for DRT was SMD: 0.27, 95% CI 0.09–0.45 (*P* = 0.003), for WB exercise SMD: 0.37, 95% CI 0.12–0.62 (*P* = 0.004) and SMD: 0.35, 95% CI 0.19–0.51 (*P* = 0.001) for the combined WB&DRT exercise. No significant differences between the types of exercise were observed (*P* = 0.822). Heterogeneity level of included trials in the WB and WB&DRT group was considerable (*I*^2^: 82.1) or substantial (*I*^2^: 63.6); but was negligible (*I*^2^: 16.5) in the DRT group (Fig. 3).

### Effect of Different Types of Exercise on TH-BMD

**Fig. 4:**
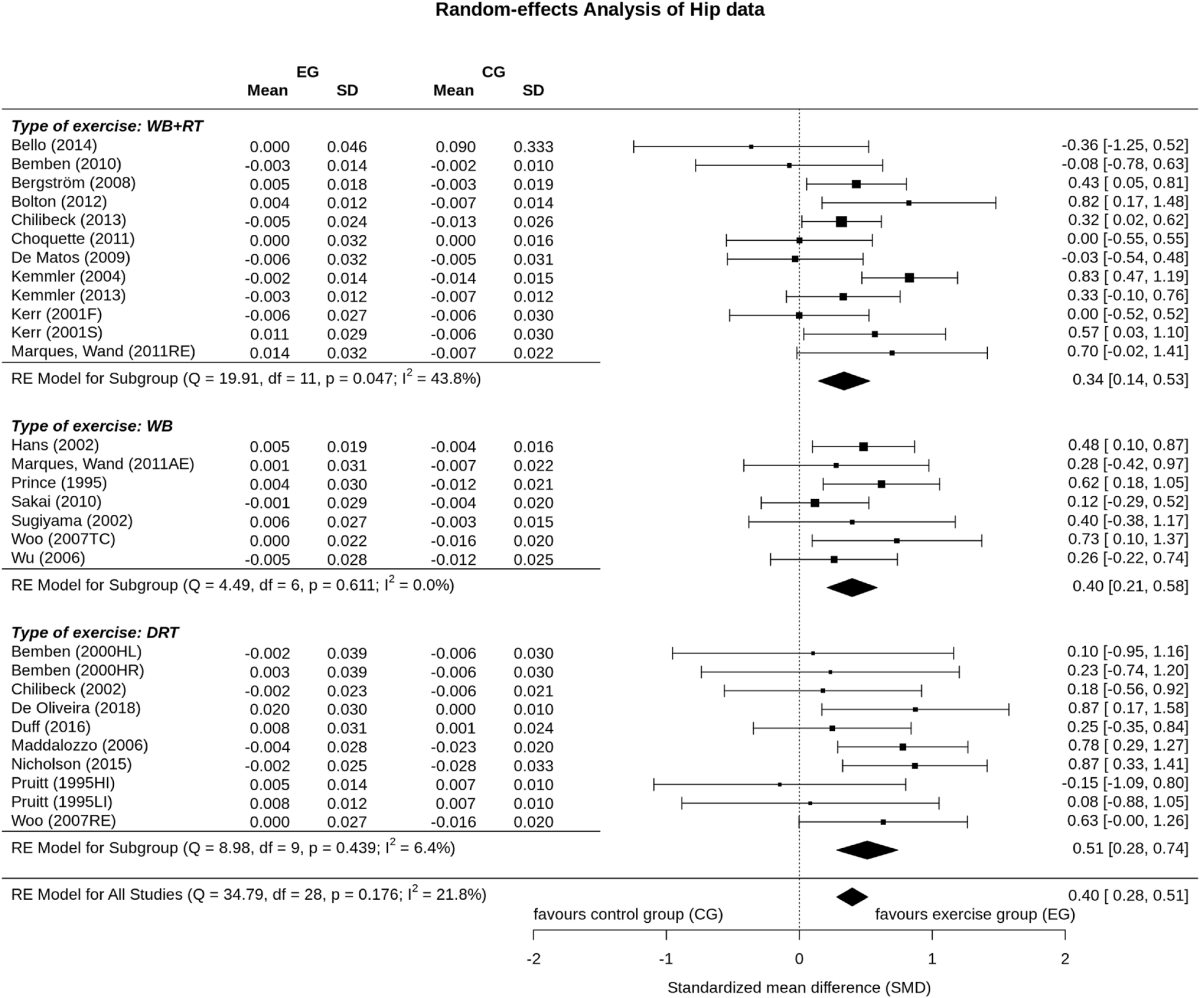
Forest plot of meta-analysis results at the total hip. The data are shown as pooled standard mean difference (SMD) with 95% CI for changes in exercise and control groups. *HI* high intensity, *LI* low intensity

Ten DRT exercise groups, seven WB exercise groups and 12 combined WB&DRT exercise groups evaluated the effect of exercise on total hip-BMD. In summary, the pooled estimate of random effect analysis for DRT was SMD: 0.51, 95% CI 0.28–0.74 (*P* < 0.001), for WB exercise SMD: 0.40, 95% CI 0.21–0.58 (*P* < 0.001) and SMD: 0.34, 95% CI 0.14–0.53 (*P* < 0.001) for the combined WB&DRT exercise. No significant differences between the types of exercise were observed (*P* = 0.554). Heterogeneity level of included trials in the WB or DRT group was negligible (*I*^2^ < 10%) and moderate (*I*^2^: 43.8%) in the WB&DRT group (Fig. 4).

Funnel plots for LS, FN and TH did not suggest positive evidence of publication bias. The regression and rank correlation test for funnel plot asymmetry did not indicate significant asymmetry for LS or TH, but did for TH with missing studies to the right (positive difference/effects). The trim and fill analysis that correspondingly imputed three studies results in a slightly higher total SMD (0.43; 95% CI 0.31–0.54) than the non-adjusted results listed in Fig. 4.

## Discussion

In this sub-analysis of a comprehensive meta-analysis, we clearly confirmed the significant positive effects of different types of exercise on BMD at LS, FN and TH in postmenopausal women. Further, WB type exercises, DRT and a combination of both types of exercise revealed at least no significant groups differences for LS, FN or TH-BMD. Thus, we verified all our hypothesis and in turn now question the data of Rahimi et al. [6]. One possible explanation for the diverging results of the present analysis and the data of Rahimi et al. [6] might be the focus on studies with women 60 years + , i.e., the advanced postmenopausal status in the latter study. Considering that menopausal transition and early menopausal status is related to considerably increased bone turnover [92, 93], there is some evidence that exercise might be more effective during early than in late post-menopause, at least with respect to trabecular bone loss [76, 94]. The meta-analysis of Shojaa et al. [3] on this issue observed only slight, non-significant differences between exercise during the early vs. late postmenopausal years,^3^ be it for LS (SMD “early”: MV = 0.64, 95% CI 0.33–0.95 vs. “late”: 0.39, 0.14–0.55) or total hip ROI (SMD: 0.51, 0.27–75 vs. 0.38, 0.20–0.56). Apart from age, both meta-analyses also differ with respect to eligibility criteria, i.e., randomization, language, publication type, medication and diseases, while the limitation on studies ≥ 6 months with healthy postmenopausal women without hormone replacement therapy and previous DRT are common to both studies. The most striking difference, on the other hand, is the low amount of studies classified into the exercise categories by Rahimi et al. [6]. Considering that only two studies were analyzed to determine the effect of WB aerobic exercise on LS-BMD (vs. *n* = 23 in the present study), one should draw definite conclusions from that data with extreme caution.

Although we consistently determined significant positive exercise effects on BMD-ROIs, (SMD: 0.26–0.51), SMDs of the single exercise trial vary substantially, particularly for the LS (*I*^2^ = 76–77%). Even in the DRT group, which can be considered as the most homogeneous group with respect to exercise type classification (see above), the heterogeneity level for LS-BMD effects was substantial (i.e., *I*^2^ > 75%). This is understandable, however, since considerable differences can be observed between the trials or study groups (Table 2) particularly with respect to exercise parameters (i.e., strain magnitude, rate [5]) and training principles (e.g., progression, periodization [5, 95]).

Revisiting the effects of different types of exercise, it is noteworthy that the effect of the WB type interventions at the LS was considerably less pronounced compared with the DRT group (SMD: 0.26 versus SMD: 0.40). This is not necessarily related to higher effects of DRT-induced direct muscular impact on LS in general, however, but to the large number of WB studies that applied low ground reaction forces (e.g., walking: *n* = 11) with corresponding axial impact loading that might not (longer) reach the LS area. Two meta-analyses [96, 97] that reported significant positive “walking effects” at FN-BMD without effects at LS-BMD support this estimation. Another surprising result is that the combined effect of WB&DRT group failed to generate relevantly higher BMD effects compared with DRT (…or apart from LS-BMD, WB type exercise). Recent evidence-based guidelines that focus on bone development [1, 4, 5] consistently recommended exercise protocols that included impact activities and progressive resistance training applied with high strain magnitude and rate. However, at this point at the latest, we have to acknowledge and discuss the limited ability of meta-analyses to derive exercise recommendations [98], largely independent of the outcome [99]. Selecting the adequate type of exercise to address a given training aim is only the first, rough decision within the training process [5, 95]. Much more challenging, particularly when addressing bone, is the consideration how to optimally specify the type of exercise in the light of the large variety of exercise parameters (e.g., strain magnitude, rate, duration, frequency, cycle number, rest periods) [5, 95]. Another modifying aspect within the exercise process is the inclusion of exercise principles [5, 95]. Applying, e.g., progression and periodization might not be important within a 10-week exercise intervention; however, considering that studies included in the present analysis on BMD average between ≥ 6 months and 30 months their relevance becomes obvious. The fact that even slight differences in exercise parameters, e.g., movement velocity of the concentric phase during DRT, significantly modify the effect on BMD [100] suggests that high complexity of exercise effects on BMD could conflict with the comprehensive meta-analytic approach. One may assume that the rather high number of study groups included in the present subgroups might even out differences at individual study levels, but this assumption is frequently wide of the mark. This might be confirmed by the considerably higher effects of DRT versus WB for TH-BMD (SMD: 0.51, 95% CI 0.28–0.74 vs. 0.34, 0.14–0.52), however, not for BMD at the adjacent FN-region (SMD-DRT: 0.27, 0.09–0.45 versus SMD-WB: 0.37, 0.12–0.62, Table 2), a constellation for which no serious explanation^4^ can be provided.

Furthermore, some limitations and study features of the present analysis may decrease the evidence and generality of our finding. (1) Although we placed high emphasis on eligibility and reliable classification of the exercise types, some decisions are certainly debatable. This may be the case for the exclusion of the study of Rhodes et al. [101]^5^ that combined non-weight bearing exercise (however only as a warm up) and DRT, while still including others (e.g., [66, 67, 87]. that applied a mixed weight bearing/non-weight bearing & DRT intervention. However, in our defense it should be noted that some studies were very lapse in their standards of exercise reporting, and so extracting the relevant information was sometimes challenging. (2) We conducted funnel plots with trim and fill analysis for the entire cohort of included studies for LS, FN and TH (not given). However, it might have been better to conduct separate funnel plots for the effects of the isolated exercise group for each ROI. On the other hand, reviewing the three funnel plots in detail, we did not observe relevant differences between the different exercise groups that might have significantly changed the present result. (3) We failed to generate reliable scores/categories for exercise intensity/strain magnitude across the different types of exercises, in order to conduct a sub-analysis for this crucial exercise parameters. A sub-analysis of our outcome adjusted for “exercise intensity/strain magnitude” might have resulted in more sophisticated results and higher overall treatment effects. (4) The present literature search was conducted up to March 1, 2019, i.e., some more studies might have been published in the meantime. However, considering the large amount of studies included in this systematic review and meta-analysis, we feel that the few additional exercise studies will not considerably modify our finding.

In conclusion, we do not share the enthusiasm for basing exercise recommendations or exercise guidelines on meta-analyses – at least in the area of “bone strengthening”. Nonetheless, at least uncritical acceptance of the acquired data should be avoided if this is done. Accurately designed randomized controlled exercise trials that manipulate a dedicated single aspect while maintaining all other exercise parameters and confounders will be more qualified to generate reliable exercise recommendations.

## Data Availability

The data that support the findings of this study are available from the corresponding author (WK), upon reasonable request.

## Footnotes

1 e.g., 10 reps at 30% maximum isometric back muscle strength [30] or 14–16 reps at 40% 1RM [18, 29]. The same might be true for some exercises of the protocol of Woo et al. [32].

2 Or two sessions of 3 min/day, 7 days/week [53].

3 Early: ≤ 8 years vs. late postmenopausal > 9 years postmenopausal for LS-BMD (LS-BMD: 40, total hip: 20 exercise groups).

4 Considering site specificity of most DRT-exercises, we would expect the opposite effect, however, differences were not significant.

5 …or Watson et al. [102]. This highly effective and time-efficient trial had to be excluded by protocol due to the number of participants (< 10%) on osteoporosis medication.
